# Editorial: Nutrition and Sustainable Development Goal 1: no poverty

**DOI:** 10.3389/fnut.2024.1466774

**Published:** 2024-08-12

**Authors:** Clinton Beckford, Ayoub Al Jawaldeh, Nur Indrawaty Lipoeto

**Affiliations:** ^1^Faculty of Education, University of Windsor, Windsor, ON, Canada; ^2^Nutrition Unit, World Health Organization, Cairo, Egypt; ^3^Department of Nutritional Sciences, Andalas University, Padang, West Sumatra, Indonesia

**Keywords:** poverty, socioeconomic, children, food and nutrition, hunger

The United Nations 2030 Agenda for Sustainable Development, launched in 2015, articulates a roadmap for sustainable global peace and prosperity. This agenda is anchored by 17 interconnected and intersectional Sustainable Development Goals (SDGs) that outline pivotal collective actions in a framework that recognizes critical synergies and symbiosis between complex factors impacting current and future human development including poverty and economic disparities, food and nutritional status, environmental integrity including climate change, health and wellbeing, educational outcomes, and technological development.

SDG 1: “No poverty” contemplates actions to “end poverty in all its forms everywhere”. This is critical given the intersections between poverty and every element of human development and quality of life, including health and wellbeing (SDG 3), education (SDG 4), productivity (SDG 8), economic inequality and disparity (SDG 5 and 10), and environmental sustainability and healthy communities (SDGs 6, 7 and 9). This Research Topic, *Nutrition and Sustainable Development Goal 1: no poverty*, discusses research that elucidates interactions between food and nutrition security and poverty at different levels, with a focus on children and communities. To start, Du et al. explore the influence of household technological advancements on rural Chinese children's nutritional intake, specifically within the context of a Chinese government initiative to increase home appliances in rural areas of the country. The study explores the repercussions of enhanced household technology on the dynamics of children's nutritional consumption patterns. The study showed that integration of household appliances, including color televisions, washing machines, and refrigerators, beneficially reshaped the nutritional consumption patterns of children, with a pronounced effect among girls. Pivotal drivers included increased parental time allocation, improved food storage capabilities, and enhanced access to information. This study provides valuable insights into potential strategies for enhancing nutritional intake among rural children and might have implications outside the Chinese context studied here.

Poverty impacts food and nutritional security, which in turn impacts health and educational performance. The review and meta-analysis by Nugroho et al. sheds light on the relationship between socio-economic status and children's working memory in four developing countries. Key findings were that poverty was associated with a lower working memory score in children and that low educational status of the mother was associated with a lower score of working memory. The key conclusion, that poverty and low educational status of the mother were significant risk factors for lowering working memory among children in developing countries, is instructive.

Household food insecurity and hunger are directly related to economic status. Low-income households experience higher rates of food insecurity and higher prevalences of hunger and hunger-related issues. Demie and Gessese addressed the lack of Ethiopian research perspectives on this topic with their mixed-methodology community-based cross-sectional household study of food insecurity in an Ethiopian town. The study found that husband or male cohabitant's occupation and wife or female cohabitant's literacy level were key factors associated with household food insecurity and hunger. The study showed that household food insecurity and hunger required urgent attention and recommended action specifically geared toward self-employed merchants in small businesses and women who are uneducated.

Poverty and nutritional insecurity disproportionately impact the most vulnerable people, including women and children. A major problem is “hidden hunger” (1, 2), characterized by inadequate nutritional intake or nutritional deficiency, which affects children and adolescents disproportionately, with serious implications for their development and cognitive abilities. The longitudinal study by Li et al. on nutritional deficiencies in countries with low sociodemographic index (SDI) aimed to provide a comprehensive estimate of the incidence of nutritional deficiency and its main subcategories at the global level and at the national level in low-SDI countries. This study also identified high-risk populations through sex and age stratification. The findings indicated high vitamin A deficiency and that protein–energy malnutrition contributed to the largest age-standardized DALY rate in 2019. Children ages 1–4 had the highest overall nutritional deficiency and dietary iron deficiency.

The research highlighted in this Research Topic is a timely look at an issue that demands ongoing attention. Global gains in the fight to eradicate extreme poverty were wiped away by the COVID-19 pandemic and the economic shocks it precipitated around the world (3). During the pandemic, extreme poverty rose after many years of sustained decline and, although poverty rates are generally back to pre-pandemic levels, low-income countries continue to lag in terms of recovery. At the same time, almost two and a half billion people experienced moderate to severe food insecurity in 2022 and over 60 percent of countries experienced rising food prices in the same year. Research about poverty and nutrition is therefore critical and this Research Topic makes an important contribution to the literature in this regard. Research within an SDG framework is also important. The 2024 SDG progress assessment indicates that we are way off course in achieving the 2023 Agenda. Stagnation is rampant on many of the 135 targets and, more alarmingly, there has been serious regression to below 2015 levels on seventeen percent of them (3). The current trajectory is, therefore, less than desirable. More research is needed to highlight this and galvanize action to turn things around in the next five and a half years.

## Author contributions

CB: Writing – original draft, Writing – review & editing. AA: Writing – review & editing. NL: Writing – review & editing.
